# Effectiveness of evidence-based medicine on knowledge, attitudes, and practices of family planning providers: a randomized experiment in Jordan

**DOI:** 10.1186/s12913-015-1101-z

**Published:** 2015-10-02

**Authors:** Marianne El-Khoury, Rebecca Thornton, Minki Chatterji, Soon Kyu Choi

**Affiliations:** International Health Division, Abt Associates Inc, Bethesda, MD USA; Department of Economics, University of Illinois at Urbana-Champaign, Urbana, IL USA; Internaltional Health Division, Abt Associates Inc, Bethesda, MD USA; The Williams Institute, University of California Los Angeles, School of Law, Los Angeles, CA USA

**Keywords:** Evidence-based medicine, Family planning, Jordan, Health providers, Biases

## Abstract

**Background:**

Provider misconceptions and biases about contraceptive methods are major barriers to family planning access for women in low- and middle-income countries, including Jordan. Evidence-Based Medicine (EBM) programs aim to reduce biases and misconceptions by providing doctors with the most up-to-date scientific evidence on contraceptive methods.

**Methods:**

We evaluate the effects of an EBM program conducted in Jordan on private providers’ knowledge, attitudes, and practices. Family planning providers randomly assigned to a treatment group were invited to attend a roundtable seminar on the injectable contraceptive Depot Medroxy Progesterone Acetate (DMPA), and to receive two 15 min one-on-one educational visits that reinforce the messages from the seminar.

**Results:**

There was low compliance with the EBM program. The study fails to detect an impact on providers’ knowledge of DMPA’s side effects or on reported clinical practices. There is suggestive evidence of a positive impact on providers’ attitudes toward and confidence in prescribing the contraceptive to their patients. There is also evidence of positive selection into program participation.

**Conclusions:**

We conclude that EBM may not be effective as a stand-alone program targeting a family planning method with a high level of provider and consumer bias. Evidence of positive selection into program participation underscores the importance of randomization to avoid overestimating the true effects of interventions.

**Trial registration:**

AEA RCT Registry, AEARCTR0000539, 11/3/2014

**Electronic supplementary material:**

The online version of this article (doi:10.1186/s12913-015-1101-z) contains supplementary material, which is available to authorized users.

## Background

Provider misconceptions and biases about contraceptive methods are major barriers to family planning access for women in low- and middle-income countries [1]. Studies in Tanzania, [2] Ghana, [3] and Bangladesh [4] have demonstrated that family planning providers sometimes deny women access to a particular contraceptive method based on their own lack of knowledge and/or biases. One approach to reduce biases and misconceptions is to provide doctors with the most up-to-date scientific evidence regarding contraceptive methods. This principle is at the core of Evidence-Based Medicine (EBM). The underlying assumption is that providers will use (and share) the evidence-based information with their patients, rather than rely on their existing beliefs regarding a particular method. This paper evaluates a program in Jordan that provided private family planning doctors with scientific evidence about Depot Medroxy Progesterone Acetate (DMPA), a hormonal injectable contraceptive with low rates of use in Jordan.

EBM has been defined as the “conscientious, explicit and judicious use of current best evidence in making decisions about the care of individual patients” [5]. The practice of EBM encourages health providers to continuously reference updated medical information and knowledge available through pre-appraised resources. By combining this external evidence with their individual clinical expertise and with patient preferences, providers are enabled to deliver high quality health services [6]. During the past decade the approach has been used with private family planning providers in Russia, [7] Armenia, [7] Bangladesh, [4] Egypt, [8] Pakistan, [8] India, [8] the Philippines, [8] and Jordan [9]. The hypothesized theory of change underlying this approach is that EBM improves knowledge of family planning methods by providing credible documented evidence, knowledge that in turn modifies existing negative attitudes and practices among health providers.

However, to our knowledge, EBM programs in low- and middle-income countries have not been rigorously evaluated. One pre-post evaluation of an EBM program, designed to provide evidence on Combined Oral Contraception (COC) pills to Jordanian doctors, found that the program increased providers’ knowledge about COC pills, their willingness to prescribe COC pills to appropriate patients, and the reported number of times they discussed family planning with their patients [9]. Elsewhere, however, experimental evaluations of EBM programs related to other interventions, such as cancer, smoking cessation, heart conditions, and pain management (all conducted in the United States), have shown mixed results [10–13].

Jordan is an appropriate setting to study the effectiveness of EBM on provider knowledge, attitudes, and practices related to family planning methods. It is a middle-income country seeking to contain its rapidly growing population, [14] yet with stagnant rates of use of modern family planning methods, partly due to pervasive provider and consumer biases and misconceptions towards these methods [15–19]. In particular, injectable contraceptives, such as DMPA, are one of the least popular contraceptive methods in the country among providers and consumers alike [18, 20]. DMPA is not a new method in Jordan and, to our knowledge, there are no market shortages or health systems constraints that explain the limited use of the contraceptive. We use an experimental study design to evaluate an EBM program in Jordan that aimed to reduce biases and misconceptions towards DMPA.

We find no significant evidence that the EBM program affected providers’ knowledge of the contraceptive’s side effects or reported clinical practices, though we find suggestive evidence of a positive impact on providers’ attitudes toward, and self-reported confidence in, discussing or prescribing DMPA. Given the low levels of demand of DMPA, and concerns about its side effects more generally among Jordanian women, providers may be resistant to adjusting their own attitudes and clinical practices, especially because the EBM approach also encourages them to take into account their patients’ preferences. We find lower rates of participation in the EBM DMPA program than prior EBM programs conducted on other contraceptive methods in Jordan. Moreover, evidence of positive selection suggests that these types of programs may not attract providers who would benefit the most from the information provided.

## Methods

### Sample

As part of a five-year program (2005–2010), the USAID-funded Private Sector Project for Women’s Health began working with private obstetricians/gynecologists and general practitioners who provide family planning services in Jordan, engaging them in multiple training programs, including EBM programs on COC and Progesterone Only Pills (POP). The EBM programs were implemented in four regions of the country: Amman, Zarqa, the South, and the North. The initial list, of 306 doctors, comprises (to the best of our knowledge) all the private obstetricians/gynecologists and general practitioners who provided family planning services in Jordan in 2011. For this evaluation, however, we chose to exclude doctors practicing in the North and South regions of Jordan, because it was logistically difficult to randomize them and to provide the treatment to a widely dispersed group. Thus we removed 23 doctors working in the North region and 15 doctors from the South region. We also excluded from the sample one doctor who had participated in the development of the EBM material. Our final sample consists of 267 private doctors who provide services in Amman and Zarqa. (See Additional file 1: CONSORT Flow Diagram.)

### Study design

Using computer generated random numbers, we randomly assigned doctors to treatment and control groups, stratifying by geographic area and gender. Of our sample of 267 private providers, 135 were randomly placed in the treatment group and 132 in the control group. Due to the stratification, the final numbers are uneven between treatment and control groups. Random assignment was conducted in 2011 prior to data collection. Participants were not informed of their treatment assignment.

### Ethics statement

Research was performed in accordance with the Declaration of Helsinki. The study received exemption from the Abt Associates Institutional Review Board (Reference number: 0600). All interviewees were asked to provide verbal consent before participating in the study and were free to decline to participate. The study posed no more than minimal risk to participants.

### Availability of supporting data

The data set supporting the results of this article are available in the Inter-university Consortium for Political and Social Research repository, http://doi.org/10.3886/E19693V1 [21]. The study protocol is available from the corresponding author, upon request.

### The EBM DMPA program

Program staff invited the treatment group doctors to attend a DMPA roundtable seminar and offered two educational visits that would reinforce the messages from the seminar. The DMPA roundtable seminar consisted of a two-hour session led by trained private peer providers who presented clinical research findings related to DMPA. The roundtable format of the seminar allowed providers to discuss these findings within the local context, with the overall goal of correcting misconceptions and biases and improving providers’ knowledge of evidence-based health benefits of method use. The seminar covered findings on return to fertility, anemia, amenorrhea, and other real or perceived side effects of DMPA. The two educational visits, to be scheduled ahead of time with individual doctors, were conducted by a trained health worker. These 15-minute one-on-one sessions, held in individual providers’ clinics, were intended to review specific information that was discussed during the seminar. These sessions had three objectives: to enhance the availability of evidence to support providers in counseling about DMPA; to address concerns about its side effects and perceived harm (such as delays in returns to fertility); and to convey information about its potential benefits (such as protective effects against cancer). The seminar and educational visits were implemented between January and June 2012.

The control group providers were not invited to a DMPA seminar and were not offered educational visits related to DMPA. However, in order to continue to engage the control group providers during the period of the study (the first six months of 2012), they were offered two repeat educational visits during this same time period; these visits were related to COC pills and a repeat of the previous year’s materials. Thus, the treatment and control groups differed in the content as well as the extent of program offerings: the treatment group was offered participation in the DMPA seminar and two DMPA educational visits, and the control group was offered two repeat educational visits on COCs. No information about DMPA was discussed during the COC visits; thus, any detectable differences on knowledge, attitudes, or practice related to DMPA can be attributed to the influence of the DMPA program.

### Data

A baseline survey was conducted in December 2011. Survey questionnaires were mailed to all 267 providers, and follow-up phone calls were made to providers who did not respond to the mailed questionnaire. The questions asked for specific information relating to the providers’ knowledge of DMPA’s side effects, their attitudes towards the method, and their clinical practices, such as discussing DMPA with clients or prescribing it. The response rate for the baseline survey was 73 % (195 providers), with no differential response rate between treatment and control group [difference: −0.009 (S.E. 0.054)]. A face-to-face endline survey with providers was conducted in January 2013. The endline survey collected information similar to the baseline: knowledge of DMPA’s side effects, attitudes towards the method, confidence about the method, and professional practices. It also collected information such as years of experience, number of patients seen, whether the provider has a dual (public and private) practice, and the types of family planning methods that the provider has prescribed in the last year. Enumerators conducted in-office surveys, yielding an endline response rate of 86 % (229 providers). There was no statistically significant differential completion rate across providers in the treatment and control [difference: −0.018 (S.E. 0.043)]. Of those who were interviewed at endline, 77 % had completed baseline interviews.

### Outcome measures

We are interested in four outcome measures: (1) providers’ knowledge of DMPA (for example, correct identification of side effects), represented as their Knowledge Score; (2) their attitudes towards DMPA (including willingness to recommend DMPA), represented as their Attitude Score; (3) their self-perceived confidence level (including confidence in discussing DMPA with clients), represented as their Confidence Score; and (4) their reported clinical practice (such as reported prescription or discussion of DMPA with clients), represented as their Practice Score. We compute scores for each of these outcome measures as described below.

To measure knowledge and attitudes towards DMPA, the survey respondents were asked to rate specific knowledge and attitudes statements using a five-point Likert scale, ranging from “strongly agree” to “strongly disagree”. Examples of these statements are: “*Use of DMPA is positively associated with weight gain*”; or, “*I would have no hesitation recommending DMPA to a healthy woman.*” We assign values ranging from −2 to +2 for each of the knowledge and attitude variables, where +2 denotes the most desirable response (e.g., “I strongly agree” that I would have no hesitation recommending DMPA to a healthy woman), and −2 denotes the least desirable response. We create a Knowledge Score and an Attitude Score for each provider using the simple average of related variables. Following Kling et al. [22] we standardize the scores by subtracting the mean and dividing by the standard deviation of the variable among the EBM control group, such that the control group’s mean is zero and its standard deviation is one.

To measure self-perceived confidence in DMPA, the respondents were asked to provide answers to three questions, using a ten-point scale. Examples of these questions are: “*How knowledgeable do you feel about DMPA?*” and “*How comfortable do you feel prescribing DMPA for your clients?*” We form the simple average of these three variables and create a Confidence Score that we also standardize according to Kling et al. [22]. Confidence outcomes were not collected at baseline.

To measure clinical practices related to DMPA, the respondents were asked to report whether they have any DMPA stock at their clinic, as well as the number of times they discussed DMPA with clients or prescribed it to their clients during the month prior to the survey. We form z-scores of each of these three variables, compute a Practice Score using the simple average, and then similarly apply Kling et al. [22] to standardize the score among the control group.

Appendix 1: Table 4 shows the components of each score and the baseline means and standard deviations, for the overall sample and by treatment and control.

### Balance across treatment and control

Random assignment is most effective at establishing comparable groups in large samples. Since ours is a small sample, we checked the balance between treatment and control groups. An examination of baseline characteristics (Appendix 2: Table 5) confirms that random assignment resulted in a sample where, on average, there are no statistically significant differences between treatment and control groups. The balance between treatment and control persists when the sample is restricted to those who completed the endline survey (not shown).

### Estimation strategy

We estimate the impact of the EBM DMPA program on outcomes (*Y*_*i*_) as follows:

1 $$ {Y}_i={\alpha}_1+{\beta}_1{T}_i+{X}^{\prime}\gamma +{\varepsilon}_{1,i} $$

T indicates that provider *i* was assigned to the treatment group; ${\beta}_1$ is the “intention to treat” (ITT) aspect of the program’s estimated impact; and ${\alpha}_1$ is interpreted as the control group’s regression-adjusted mean outcome. When we regress the standardized outcome scores on T, the resulting coefficient estimates are the mean effect sizes and capture the average impact in terms of standard deviations of the outcome variables in the control. The standardization facilitates comparison of impact magnitudes across outcomes. The regression includes a vector *X* of baseline covariates, which serves to reduce noise attributable to random differences between the groups. We impute missing baseline data with the mean values and include dummy variables to the regression to indicate imputation. Because of missing baseline data, we show results both with and without the baseline covariates.

To adjust for non-compliance, we also estimate the local average treatment effects of actual participation in the complete program (defined as attending the seminar and receiving both of the educational visits). To do this, we instrument compliance with the EBM program (endogenous variable) with the treatment assignment, using two-stage least squares (2SLS) (Eq 2 and 3). This approximates the “treatment on the treated” (TOT) effects (${\beta}_2)$, which are expected to be larger than the ITT estimates.

2 $$ {Y}_i={\alpha}_2+{\beta}_2\kern0.5em AC\widehat{TUA}L\_{T}_i+{\varepsilon}_{2,i} $$

The first stage specification is given by:

3 $$ ACTUAL\_{T}_i={\alpha}_3+{\beta}_3{T}_i+{\varepsilon}_{3,i} $$

One potential threat to the validity of the specifications is the risk of spillovers from treatment to control; however, this is unlikely for a number of reasons. First, providers in the sample have their own private clinics and do not share practices. Second, the surveys asked whether providers had discussed medical evidence with other providers or medical professionals, and we find no statistically significant differences in frequency of discussion between treatment and control groups.

## Results

**Fig. 1:**
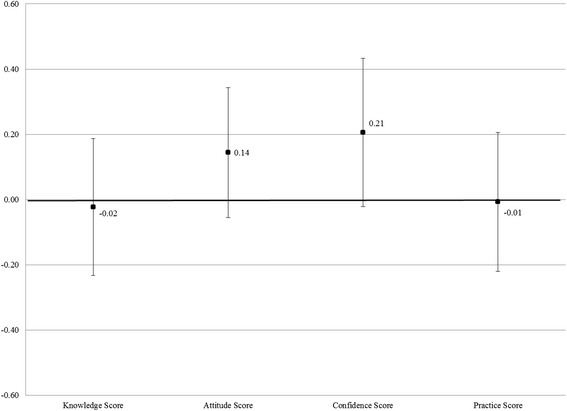
Impact of EBM program on provider outcomes. The black squares denote the Intent-To-Treat coefficients without the baseline covariates; The error bars denote the 90 % confidence level; Robust standard errors; For all scores, the control group mean is zero and the standard deviation is one; The Y-axis is measured in terms of standard deviations of the outcome variable in the control group. *N* = 229 providers

Figure 1 illustrates the results from separate regressions of the ITT impact estimates of the EBM program, excluding baseline covariates. The black squares represent the value of the coefficient on the treatment indicator for each outcome variable. A positive coefficient indicates that the program had a positive effect on outcomes of interest, such as knowledge about DMPA or attitudes towards the method. The figure also shows the 90 % confidence intervals for each estimated coefficient. When the confidence interval contains the value “0.00,” we are unable to reject the null hypothesis that there was no significant difference between the treatment and control.

Figure 1 shows no significant impact of the EBM program on knowledge of DMPA’s side effects or on reported clinical practices. The point estimates on the Knowledge and Practice Scores are −0.02 and −0.01 respectively. Figure 1 also shows that the Attitude and Confidence Scores of providers in the treatment group are 0.14 and 0.21 standard deviations higher (respectively) compared to the control group. While they suggest a move in the positive direction, these differences are not statistically significant at traditional confidence levels.

Table 1 shows the ITT estimates when adding covariates in the regressions and imputing missing baseline data. The impact estimates on the treatment indicator are slightly smaller in magnitude than the ones in the parsimonious specification without covariates. Coefficients on the Attitude and Confidence Scores remain positive (0.12 and 0.20 standard deviations in magnitude, respectively), but are not statistically significant. Baseline Attitude Scores and Practice Scores are strongly and positively associated with key outcomes at endline. At the same time, there is no relationship between the baseline Knowledge Score and any outcome measure. Note that we are powered to detect a difference of 0.33 standard deviations between the treatment and control groups for our Knowledge, Attitudes, Confidence and Practice Scores, using conservative assumptions (alpha 0.05, power 0.90, covariates explaining 0.25 of the variation).

**Table 1:** ITT impact of EBM program on outcomes (including baseline covariates)

	(1)	(2)	(3)	(4)
	Knowledge score	Attitude score	Confidence score	Practice score
Independent variables				
Treatment	−0.03	0.12	0.20	0.04
	[0.13]	[0.13]	[0.14]	[0.12]
Female	0.67***	−0.10	0.17	0.24**
	[0.13]	[0.13]	[0.16]	[0.11]
Amman	0.05	0.04	0.24	0.23
	[0.15]	[0.14]	[0.17]	[0.15]
Number of patients	−0.000	0.000	0.001	0.003***
	[0.001]	[0.001]	[0.001]	[0.001]
Baseline knowledge score	0.07	0.02	0.03	0.15**
	[0.08]	[0.07]	[0.08]	[0.07]
Baseline attitude score	0.13*	0.27***	0.27***	0.08
	[0.08]	[0.09]	[0.08]	[0.06]
Baseline practice score	0.06	−0.02	0.10**	0.34***
	[0.07]	[0.05]	[0.05]	[0.10]
Constant	-0.38*	0.04	−0.36	-0.61***
	[0.20]	[0.2]	[0.24]	[0.20]
N	229	229	229	229
R-squared	0.20	0.14	0.18	0.33

Each column shows results from a separate OLS regression. Missing vales at baseline are imputed. All regressions include dummies for missing variables (coefficients not shown). ***Significant at the 1 percent level. **Significant at the 5 percent level. *Significant at the 10 percent level

Not all of the providers in the treatment group participated in the EBM seminar suggesting that the ITT estimates above are a lower bound of the estimated treatment effects. Approximately 45 % of the treatment group providers invited to the EBM seminar participated in the seminar (Table 2). Two control group providers attended the seminar. Approximately 82 % of treatment group providers received the first educational visit, while 79 % received the second educational visit; 76 % of the treatment group providers received both visits. According to the program monitoring data, the provider’s busy schedule was the most commonly cited reason for not participating in the seminar or the educational visits. Overall, 38 % of the treatment group received both educational visits and attended the seminar.

**Table 2:** Compliance with the EBM program (percentages)

	Treatment	Control
Providers who attended the EBM seminar	0.45	0.01
Providers who received first educational visit on DMPA	0.82	0
Providers who received second educational visit on DMPA	0.79	0
Providers who received both educational visits on DMPA	0.76	0
Providers who received at least one educational visit on DMPA	0.85	0
Providers who attended the seminar AND received both educational visits on DMPA	0.38	0

*N* = 135 for treatment; *N* = 132 for control

**Fig. 2:**
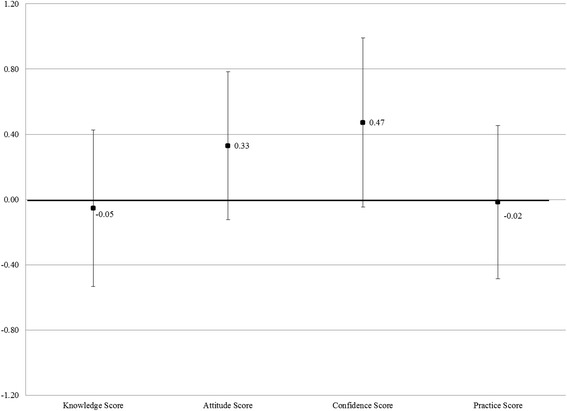
Adjusted impact of EBM program on provider outcomes (TOT estimates). The black squares denote Treatment on the Treated coefficients from Equation (2); The error bars denote the 90 % confidence level; Robust standard errors. For all scores, the control group mean is zero and standard deviation is one. *N* = 229 providers

We show the TOT estimates in Fig. 2. The TOT estimates are robust to various (partial) specifications of the endogenous variable, such as participation in the seminar and at least one educational visit rather than participation in the entire EBM program (not shown). While larger than the ITT estimates in magnitude, the estimates are not statistically significant.

We find some evidence of positive selection into the seminar (Table 3). A comparison of baseline characteristics between treatment group providers who did and did not participate in the seminar shows that providers who attended the seminar were, on average, significantly more knowledgeable initially on DMPA than those who did not attend: the average baseline Knowledge Score for those who attended the seminar is 0.47 standard deviations higher. Providers who attended the seminar are also more likely to have more positive attitudes, though these estimates are not statistically significant.

**Table 3:** Baseline statistics, by seminar attendance

	Attended seminar	Did not attend seminar	Difference (A) - (B)	
			Mean	SE
	(A)	(B)		
Baseline Knowledge Score^(a)^	0.47	0.00	0.47**	0.21
Baseline Attitude Score^(a)^	0.21	0.00	0.21	0.20
Baseline Practice Score^(a)^	0.10	0.00	0.10	0.21
Female	0.77	0.61	0.16**	0.08
Amman	0.84	0.78	0.05	0.07
Years of family planning experience	17.0	17.3	−0.29	1.57
Sample range^(b)^	46–61	50–74		

^(a)^For all scores, the group of providers who did not attend the seminar has a mean of zero and a standard deviation of one

^(b)^The sample size ranges from 46 to 61 in column (A) and from 50 to 74 for column (B) because of missing observations

**Significant at the 5 percent level.

## Discussion

This experimental study measures the impact of an EBM program in Jordan aimed at addressing private provider biases and misconceptions towards an unpopular contraceptive method, DMPA.

We fail to detect an impact of the EBM program on knowledge. However, we find suggestive evidence that the program led to improved attitudes and confidence. Although we hypothesized that the program would increase knowledge which would then improve attitudes, our results do not support this hypothesis. We also fail to detect a change in provider-reported practices related to DMPA—such as stocking DMPA, discussing DMPA with patients, and prescribing DMPA. Strong consumer bias against this method may also have made it difficult for providers to discuss or recommend this method. Our findings challenge the theory of behavior change that underlies the EBM approach. It is unclear whether the EBM approach as designed in this program is appropriate or sufficient in bringing about changes in provider behavior. In this context, EBM may need to be coupled with additional consumer targeted measures that directly address barriers against DMPA use.

Compared to participation rates at prior EBM seminars conducted in Jordan for other family planning methods (71 and 66 % for POP and COC, respectively), attendance at the EBM DMPA seminar was very low at 45 %. While low attendance may be due to provider fatigue, it may also be linked to the low demand and negative provider and consumer attitudes towards DMPA compared to other family planning methods. We find evidence of self-selection into the roundtable seminar: treatment providers who attended the DMPA seminars were on average better informed and more positive toward DMPA than treatment providers who did not attend. This finding underscores the importance of conducting experimental evaluations to evaluate this type of program. Analyses using cross-sectional data are likely to be biased upward.

The results from this study are quite different from the earlier pre-post EBM COC evaluation. That study provided suggestive evidence that EBM increased provider knowledge about COC pills as well as their willingness to prescribe COC pills to appropriate clients and the number of times they discussed COC with their patients. It is possible that EBM is a promising approach for addressing medical topics where there is less pre-existing provider bias. Alternatively, it is possible that the providers who participated in the EBM COC seminars may have had high levels of knowledge and fewer biases against COCs initially, given that this study was a pre-post and did not have an experimental design.

Given that we surveyed the majority of private providers offering family planning services in Amman and Zarqa, our results are generalizable in these particular areas. Our results, however, may not be applicable to EBM programs implemented elsewhere, or those related to other family planning methods or topics.

Our study has some important limitations. The relatively small sample size limits our ability to detect small to medium effects with statistical precision. The questions asked in the survey may be insufficient to comprehensively gauge health provider attitudes. A more detailed set of questions would have been preferable; however the questionnaires were designed to be concise to minimize burden on the respondents and remain within budget. In addition, reported practices can differ substantially from actual practices [23, 24]. It would have been ideal to measure provider practices through the use of a mystery client survey or client exit surveys. However, these options were not socially acceptable in this particular setting, and risked creating disruptions in the relationship between providers and program staff. Finally, with only one seminar and two follow-up educational visits over the course of six months, the dosage of this intervention may be too low to alter attitudes and clinical behaviors. We are unable to determine with our study design whether a more intensive approach would have been more effective.

## Conclusions

Our study does not find evidence that EBM is effective as a stand-alone program targeting a highly unpopular family planning method in a middle-income country.

The study suggests that non-randomized methods are inadequate for measuring the impact of programs with this type of self-selection. If EBM is to be used widely in conjunction with family planning methods in low- and middle-income countries, it should be rigorously evaluated again with a different family planning method and/or in conjunction with a demand-side campaign, possibly in different settings. An evaluation would also benefit from a mixed method approach as qualitative data could help better contextualize and explain findings. As provider misconceptions and biases towards family planning methods continue to create barriers to access among women in low- and middle-income countries, the family planning community needs to continue to implement and test interventions that address this issue.

## Appendix 1

**Table 4:** Outcome measures at baseline

	Overall sample			Treatment			Control		
	Mean	S.D.	N	Mean	S.D.	N	Mean	S.D.	N
**Knowledge of DMPA** ^**a**^									
Women are at a higher risk of ectopic pregnancy if they use DMPA long term	0.77	0.93	192	0.72	0.96	96	0.81	0.90	96
DMPA use is associated with an increased incidence of breast cancer	0.85	0.97	193	0.85	1.03	96	0.85	0.92	97
Women who use DMPA are less likely to suffer from anemia	0.64	0.98	193	0.77	0.92	96	0.52	1.02	97
From time to time, a woman using DMPA should give her body a rest	−0.18	1.26	192	0.06	1.28	96	−0.42	1.19	96
Use of DMPA is positively associated with weight gain	0.09	1.01	192	0.04	1.00	96	0.15	1.02	96
DMPA use is safe for most healthy women	1.13	0.77	191	1.16	0.87	95	1.10	0.66	96
Women who use DMPA are more likely to experience amenorrhea^b^	---	---	---	---	---	---	---	---	---
Women who use DMPA are more likely to experience spotting^b^	---	---	---	---	---	---	---	---	---
**Attitudes towards DMPA** ^**a**^									
For some women, amenorrhea can be a benefit	0.63	1.10	191	0.79	1.04	95	0.48	1.13	96
I should not prescribe DMPA to nulliparous women who wish to delay childbirth	−0.65	1.24	191	−0.57	1.28	94	−0.72	1.21	97
If women in Jordan had more information about DMPA, more women might accept its use	0.85	1.05	182	0.76	1.11	88	0.93	0.99	94
I would have no hesitations to recommend DMPA to a healthy woman who wanted to use this method	0.95	1.04	192	0.98	0.99	96	0.93	1.09	96
**Perceived confidence towards DMPA** ^**b**^									
How knowledgeable do you feel about DMPA?	---	---	---	---	---	---	---	---	---
How confident do you feel discussing DMPA with clients?	---	---	---	---	---	---	---	---	---
How comfortable do you feel prescribing DMPA as a contraceptive method to your clients?	---	---	---	---	---	---	---	---	---
**Baseline Practices**									
Availability of DMPA stock at clinic (binary)	0.22	0.41	183	0.20	0.40	94	0.24	0.43	89
Average # times discussed DMPA with clients in past month	5.41	7.57	187	5.10	5.76	94	5.73	9.06	93
Average # times prescribed DMPA to clients in past month	2.19	3.62	184	2.00	3.49	91	2.38	3.75	93

^a^Values range from −2 to +2, where +2 denotes the most knowledgeable or desirable response and −2 denotes the least knowledgeable or desirable response

^b^These outcomes were not collected at baseline

## Appendix 2

**Table 5:** Characteristics of providers

	Treatment	Control	Difference (T-C)	
			Mean	SE
Female^(a)^	0.68	0.69	−0.01	0.06
Average years of clinical experience	24.6	24.8	−0.20	1.07
Average years of clinical experience in family planning	17.1	17.6	−0.50	1.19
Providers with dual practice (in public and private sectors)	0.14	0.09	0.05	0.04
Method provision/prescription in clinic				
Copper intrauterine contraceptive device (IUCD)	0.93	0.95	−0.02	0.03
Hormonal intrauterine system (Mirena®)	0.67	0.62	0.06	0.06
Implant (Implanon®)	0.32	0.29	0.03	0.06
Combined oral contraceptive (COC) pill	0.97	0.99	−0.02	0.02
Progestin-only pill	0.94	0.95	−0.01	0.03
Vaginal ring (NuvaRing®)	0.41	0.39	0.02	0.06
Condom	0.94	0.97	−0.03	0.03
Female sterilization (e.g. bilateral tubal ligation)	0.57	0.56	0.01	0.07
Male sterilization (vasectomy)	0.08	0.09	−0.01	0.04
Baseline Knowledge Score (standardized)^(b)^	0.18	0.00	0.18	0.15
Baseline Attitude Score (standardized)^(b)^	0.15	0.00	0.15	0.15
Baseline Practice Score (standardized)^(b)^	−0.15	0.00	−0.15	0.12
Baseline Availability of DMPA stock at clinic^(b)^	0.20	0.24	−0.03	0.06
Baseline Average # times discussed DMPA with clients in past month^(b)^	5.1	5.7	−0.64	1.10
Baseline Average # times prescribed DMPA in past month^(b)^	2.0	2.4	−0.38	0.53
N	117	112		

^(a)^ N = 137 for the control group; N = 135 for the treatment group

^(b)^ The sample size ranges from 89 to 96 for the control group and from 91 to 97 for the treatment group because of missing observations

## Additional file

Additional file 1:
**Flow diagram that shows the final sample of the study.** Diagram style adapted from CONSORTStatement.org (http://www.consort-statement.org/consort-statement/flow-diagram). (TIFF 204 kb)

## Abbreviations

COC: Combined Oral Contraception
CONSORT: Consolidated Standards of Reporting Trials
DMPA: Depot Medroxy Progesterone Acetate
EBM: Evidence-Based Medicine
ITT: Intention to treat
POP: Progesterone Only Pills
SHOPS: Strengthening Health Outcomes through the Private Sector Project
TOT: Treatment on the treated
USAID: United States Agency for International Development
