# Supplementary figures and images for: Effectiveness of evidence-based medicine on knowledge, attitudes, and practices of family planning providers: a randomized experiment in Jordan
Source: BMC Health Serv Res. 2015 Oct 2;15:449. doi: 10.1186/s12913-015-1101-z (PMC4592549; doi:10.1186/s12913-015-1101-z)

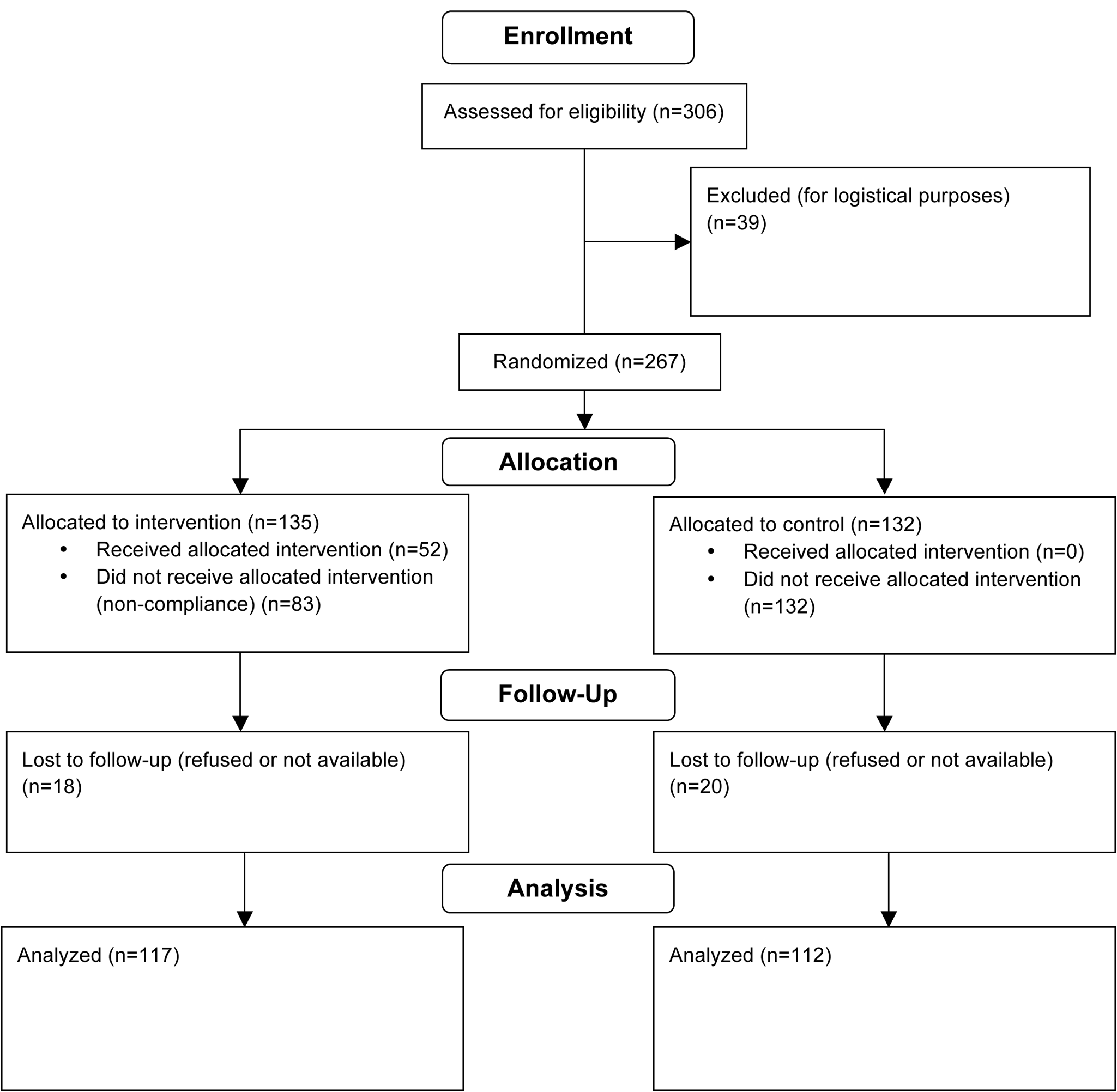

Supplement: Additional file 1: — Flow diagram that shows the final sample of the study. Diagram style adapted from CONSORTStatement.org (http://www.consort-statement.org/consort-statement/flow-diagram). (TIFF 204 kb) [file 12913_2015_1101_MOESM1_ESM.tiff]
